# Editorial: Fusion proteins for the detection of pathogens or pathogen receptors

**DOI:** 10.3389/fbioe.2025.1660729

**Published:** 2025-07-29

**Authors:** Roger M. Benoit

**Affiliations:** Laboratory for Multiscale Bioimaging, PSI Center for Life Sciences, Villigen, Switzerland

**Keywords:** fusion proteins, protein sensors, inteins, protein labeling, pathogen research, electron-dense protein tags, protein design, ultrastructure

## Introduction

Menaces from pathogens, such as viruses, prions, bacteria and fungi (and their toxins), are widespread. Novel tools for the detection of pathogens and their host receptors are of critical importance for improving our understanding of the molecular mechanisms underlying diseases and for developing fast, affordable, and accurate diagnostics.

Recombinant DNA technology has enabled the genetic fusion of DNA fragments coding for different proteins and linkers, to produce single-chain fusion proteins comprising multiple functionalities. The simplest engineered fusion proteins are composed of a tag or fluorescent protein, an optional linker with specific properties (e.g., flexible, rigid, or containing a protease recognition site) and a protein domain of interest. As an example, a fusion protein comprising a fluorescent protein, connected via a flexible linker to the receptor-binding domain of SARS-CoV-2 (Bierig et al., 2020), can be used as a tool to visualize the SARS-CoV-2 receptor angiotensin-converting enzyme 2 (ACE2) on cells in fluorescent microscopy. Examples of more complex engineered proteins include biosensors that can detect changes in mammalian cell lines, for example, aspartate levels (Davidsen et al., 2024), or biosensors for the detection of specific bacterial second messengers (Kaczmarczyk et al., 2024).

Engineered protein-based sensors hold promise to enable efficient detection or monitoring of a wide range of pathogens and pathogen-induced changes in host cells.

## Research Topic

The aim of this Research Topic was to collect articles focused on fusion proteins that enable new possibilities for pathogen research and diagnosis. The Research Topic comprises three articles presenting original research, and one review article.

The properties of linkers in fusion proteins are often critical for achieving a desired function. While flexible linkers allow freedom of motion between two connected domains, rigid linkers (Collu et al., 2022; Jeong et al., 2016; Kwon et al., 2020) can improve accessibility of specific protein regions.

**FIGURE 1:**
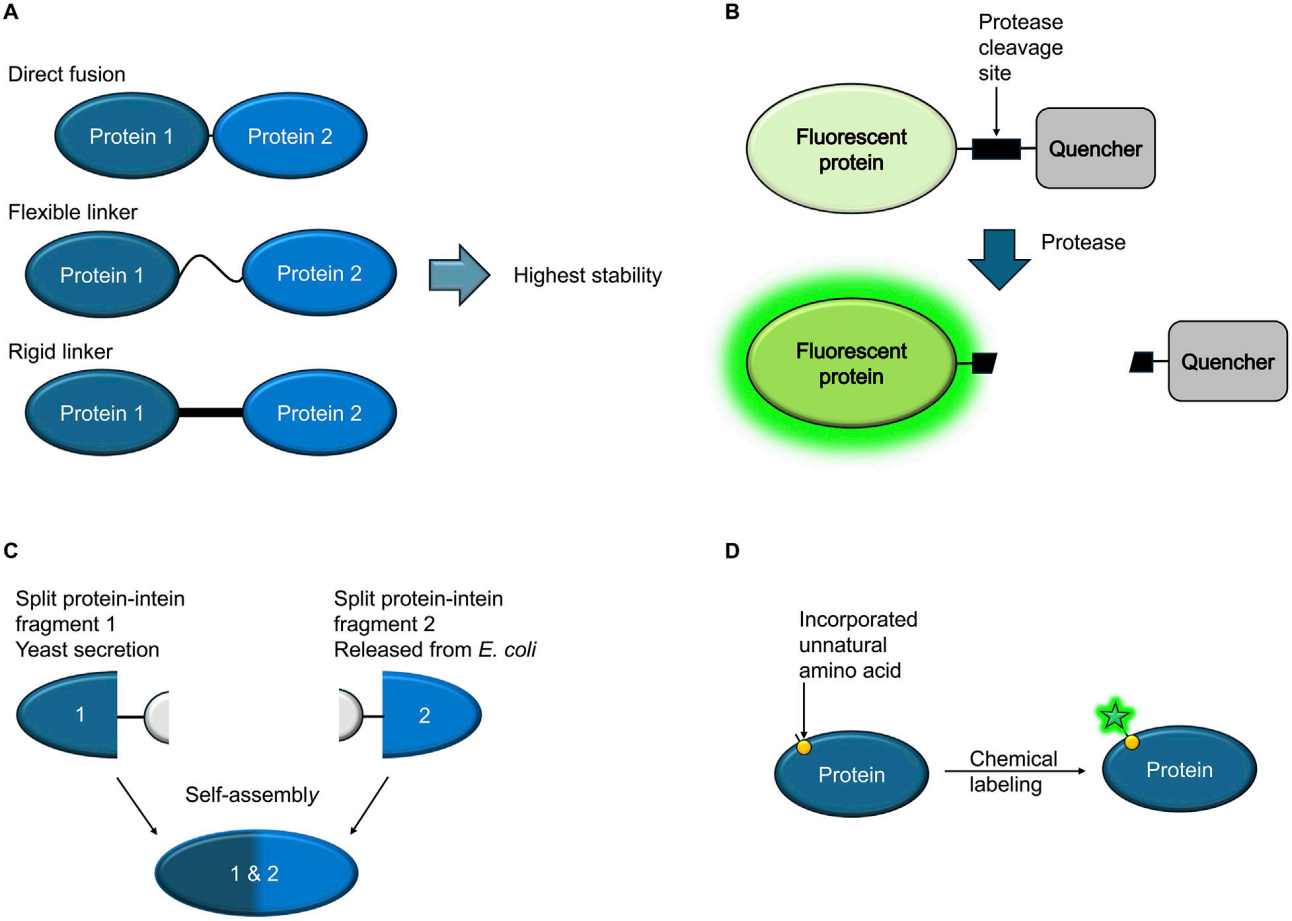
Schematic overview **(A)** Hybrid proteins consisting of two different vaccine candidate proteins, genetically fused with or without a linker, constitute a feasible alternative to available vaccines. The composition of the linker between the two proteins can affect the stability and biological activity of the fusion proteins. **(B)** A fluorescent protein is genetically fused to a quencher via a linker comprising a protease cleavage site that is recognized by a specific protease from a pathogen. If exposed to a solution containing the protease, the quencher is uncoupled from the fluorescent protein, resulting in increased fluorescence. **(C)** Inteins can be used to express two protein segments separately, followed by self-assembly into a complete, functional protein. The assembly can take place *in situ* in the culture medium. **(D)** Incorporation of unnatural amino acids displaying bioorthogonal handles on the protein surface enables subsequent selective chemical labeling with small molecule dyes that are less bulky than fluorescent proteins. Labeling can be carried out in live cells.

Zane et al. describe their work on genetic fusions comprising pneumococcal surface protein A (PspA) and detoxified pneumolysin (PdT) to develop improved vaccines against the bacterial pathogen *Streptococcus pneumoniae*. Their data show the importance of linker composition and length for the stability of fusion proteins for medical applications (Figure 1A).

Devoy et al. engineered a modular protease sensor that enables detection of the presence of proteases using a cell-free setting. Specific protease activities can be used as biomarkers to detect viruses or cancer. Overall, the sensor consists of a green fluorescent protein (GFP), a protease cleavage site, a quencher and two different tags, one at the N-terminus, the other at the C-terminus. A specific protease, matching the chosen recognition/cleavage site in the construct, hence cleaves off the quencher, resulting in an increase in fluorescence, providing a simple readout (Figure 1B). Another possible readout is provided through the uncoupling of the two tags upon protease cleavage.

Typically, fusion proteins consist of a single continuous protein chain resulting from the expression of genetically fused elements. Inteins (protein introns) make it possible to express two protein segments separated by an intervening sequence, followed by autocatalytic splicing out of the intein part, resulting in ligation of the two flanking proteins (Lennon and Belfort, 2017). In this Research Topic, Wang et al. describe the use of intein-based splicing of proteins *in situ* by engineered microbial consortia. By secreting one intein-fused protein fragment from yeast cell cultures and releasing a matching complementary domain from co-cultured bacterial cells using an autolysis system, extracellular reconstitution takes place directly in the culture (Figure 1C). Possible uses include modular production of functional proteins, as well as logic computation or antibiotic resistance engineering.

While fusion protein technology often allows the engineering of valuable tools for the detection of pathogens or their receptors, there are also applications that pose technical challenges. For example, bulky fluorescent proteins can disrupt proper secretion of effectors. Singh and Kenney in their review article outline available bacterial protein labeling strategies in the context of host-pathogen interactions in detail and discuss novel approaches for the visualization of bacterial proteins and host-pathogen interactions that can overcome such problems. For example, genetic code expansion enables the incorporation of bioorthogonal handles into the protein of interest, allowing subsequent chemical labeling with dyes, even in live cells (Figure 1D).

## Future directions

The study of pathogens and their interaction with host receptors is an active field of research. Novel tools, such as the strategies described in this Research Topic, play an important role towards enabling detection of specific proteins, or towards optimizing processes for the identification and characterization of pathogen-induced disorders.

Structural information can be highly useful for the design of fusion proteins, allowing a precise choice of domain boundaries. Natural proteins can furthermore be used as a guide on how to design or link proteins [molecular biomimetics, (e.g., Collu et al., 2022)].

Computational structure prediction and *de novo* protein design have recently come of age (e.g., Baek et al., 2021; Jumper et al., 2021; Kortemme, 2024; Pacesa et al., 2025) and their use for the design and optimization of constructs, linkers, and binders will likely become widespread.

Another current trend in structural research is the aim to solve structures of proteins and protein complexes not in their isolated form, but as much as possible in their native environments. *In situ* structural biology methods such as correlated light and electron microscopy (CLEM) (de Boer et al., 2015) or X-ray based methods such as holographic nano-tomography (Kuan et al., 2020) or ptychography (Bosch et al., 2024) allow new insights into cellular ultrastructure. Challenges include low contrast or the localization of proteins of interest in the crowded cellular environment. There is a need for novel tools, such as electron-dense tags like ferritin (Clarke and Royle, 2018) that can increase contrast or reveal the localization of specific cellular features. Fusion protein tools have a high potential to enable new possibilities in this field for pathogen research as well as for other areas of research.
